# DNA metabarcoding reveals diverse diet of the three-spined stickleback in a coastal ecosystem

**DOI:** 10.1371/journal.pone.0186929

**Published:** 2017-10-23

**Authors:** Eglė Jakubavičiūtė, Ulf Bergström, Johan S. Eklöf, Quiterie Haenel, Sarah J. Bourlat

**Affiliations:** 1 Nature Research Centre, Vilnius, Lithuania; 2 Department of Aquatic Resources, Institute of Coastal Research, Swedish University of Agricultural Sciences, Öregrund, Sweden; 3 Department of Ecology, Environment and Plant Sciences, Stockholm University, Stockholm, Sweden; 4 Zoological Institute, University of Basel, Basel, Switzerland; 5 Department of Marine Sciences, University of Gothenburg, Gothenburg, Sweden; University of Guelph, CANADA

## Abstract

The three-spined stickleback (*Gasterosteus aculeatus* L., hereafter ‘stickleback’) is a common mesopredatory fish in marine, coastal and freshwater areas. In large parts of the Baltic Sea, stickleback densities have increased >10-fold during the last decades, and it is now one of the dominating fish species both in terms of biomass and effects on lower trophic levels. Still, relatively little is known about its diet—knowledge which is essential to understand the increasing role sticklebacks play in the ecosystem. Fish diet analyses typically rely on visual identification of stomach contents, a labour-intensive method that is made difficult by prey digestion and requires expert taxonomic knowledge. However, advances in DNA-based metabarcoding methods promise a simultaneous identification of most prey items, even from semi-digested tissue. Here, we studied the diet of stickleback from the western Baltic Sea coast using both DNA metabarcoding and visual analysis of stomach contents. Using the cytochrome oxidase (CO1) marker we identified 120 prey taxa in the diet, belonging to 15 phyla, 83 genera and 84 species. Compared to previous studies, this is an unusually high prey diversity. Chironomids, cladocerans and harpacticoids were dominating prey items. Large sticklebacks were found to feed more on benthic prey, such as amphipods, gastropods and isopods. DNA metabarcoding gave much higher taxonomic resolution (median rank genus) than visual analysis (median rank order), and many taxa identified using barcoding could not have been identified visually. However, a few taxa identified by visual inspection were not revealed by barcoding. In summary, our results suggest that the three-spined stickleback feeds on a wide variety of both pelagic and benthic organisms, indicating that the strong increase in stickleback populations may affect many parts of the Baltic Sea coastal ecosystem.

## Introduction

The three–spined stickleback (*Gasterosteus aculeatus* L., hereafter ‘stickleback’) is a common mesopredatory fish of high ecological interest, widespread all over the northern hemisphere in various habitats including coastal seas, estuaries, freshwater lakes and streams [1]. The stickleback is also an eco-genomic model organism, well-studied in terms of behavioural and evolutionary ecology [2–4]. Knowledge on the role of sticklebacks in aquatic food webs is, however, rather limited, especially in coastal and marine areas. To better understand the ecological role of sticklebacks, their feeding patterns and diet preferences need to be described, as feeding behaviour may affect community composition and food web functions.

In the brackish Baltic Sea, stickleback abundance has increased more than 10-fold during the last decade [5]. Currently, it constitutes up to 10% of the planktivorous biomass in offshore areas [5,6], and dominates fish assemblages in some coastal areas during summer, when adults immigrate from the open sea to spawn [6–8]. Experiments and field surveys indicate that sticklebacks may alter coastal food webs by feeding on and influencing lower trophic levels (e.g. grazers) [9], and worsening the effects of nutrient enrichment through cascading effects that increase the biomass of filamentous algae [6,7,10,11]. Moreover, sticklebacks may suppress populations of large predatory fish, such as northern pike and Eurasian perch, by predation on eggs and larvae, and the intraguild predation between sticklebacks and these large predatory fish may contribute to destabilizing food webs [5,12,13]. Thus, the increasing abundances of sticklebacks, in combination with their central role in ecosystem functioning, points to the need for more detailed knowledge on stickleback diets.

However, fish diet studies are challenging, with different methods having their own set of limitations. For the last century, the standard has been to visually identify prey from stomach contents, based on prey morphology [14]. This time-consuming method relies heavily on taxonomic expertise and can only be done when prey organisms are not too digested. Because most prey organisms rapidly degrade in stomachs, a high taxonomic resolution is often not possible, and a significant share of the prey tissue in the guts is often unidentifiable (e.g., [15]). A highly promising alternative to visual prey identification is metabarcoding methods, which combine DNA-based identification and high-throughput DNA sequencing, using taxonomically broad PCR primers to mass-amplify DNA barcodes from bulk samples (such as environmental samples or gut contents) [16]. Metabarcoding enables the identification of most prey items, even when diets are broad and diverse [17], and the simultaneous analysis of many samples. The aim of this study was to investigate the diet of the three-spined stickleback in coastal areas of the western central Baltic Sea, using a combination of classic (visual) and emerging (DNA metabarcoding) methods. Specifically, we addressed three questions: 1) what do sticklebacks eat in coastal areas, 2) how does stickleback diet depend on its body size, and 3) how do visual and DNA-based methods compare in terms of prey identification from stomach content. Accurate diet determination will provide more comprehensive information on coastal food webs, knowledge which is highly relevant in the context of ecosystem-based management to assess and potentially counteract the undesirable effects of massive increases of sticklebacks on the ecosystem [10,18].

## Material and methods

This study was made in accordance with the ethical regulation laid down in the Swedish ordinance SJVFS 2012:26, which is the Swedish implementation of the Directive 2010/63/EU of the European Parliament and of the Council on the protection of animals used for scientific purposes. The fish died in the process of lifting the nets; after sticklebacks were removed from the nets they were immediately put in 95% ethanol. The fish sampling procedures applied in the project were also judged and approved by the Ethical Board on Animal Experiments of the County Court of Uppsala, Sweden, permit C 139/13.

### Study sites and sample collection

**Fig 1:**
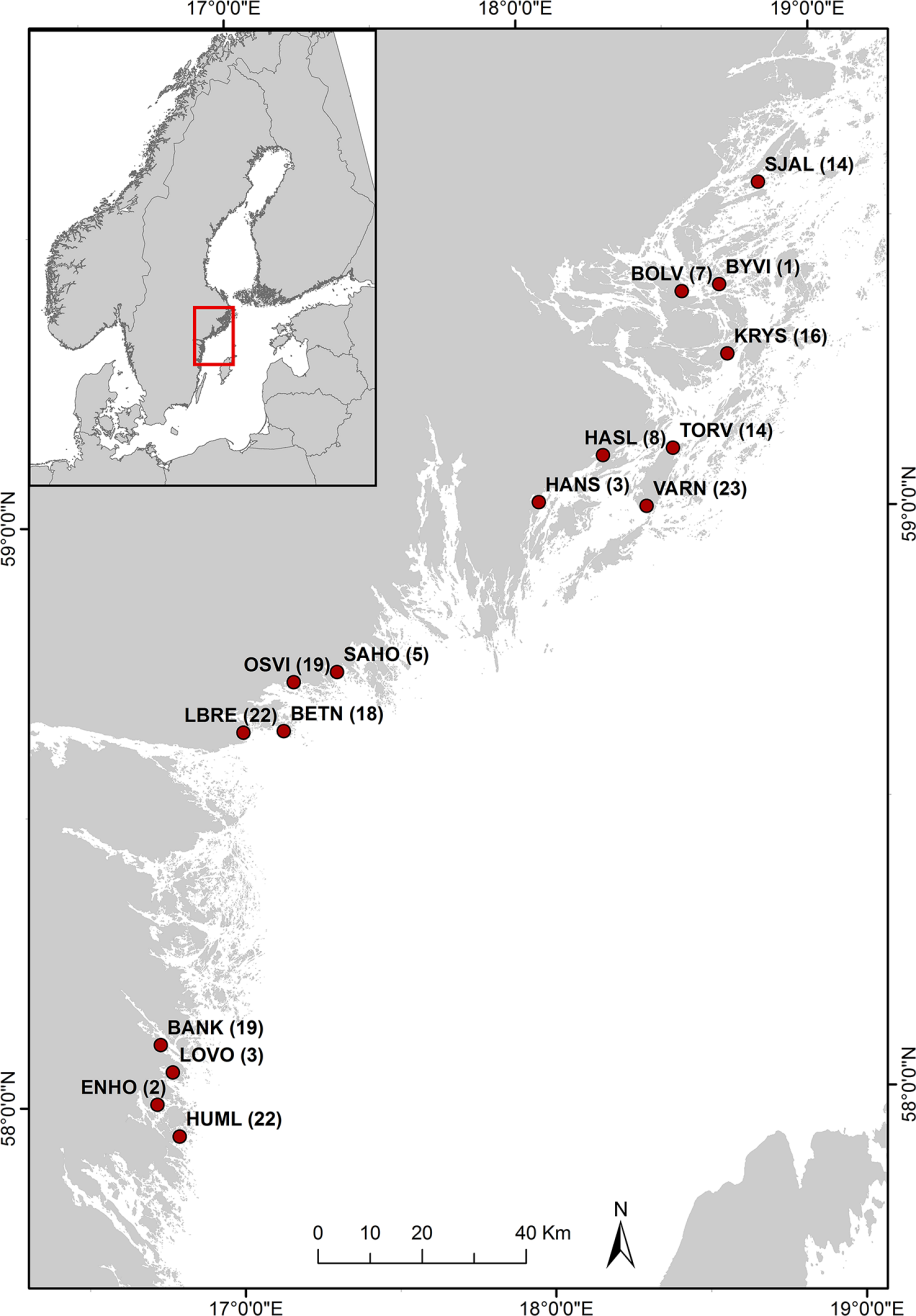
Sampling sites. Numbers in brackets indicate number of sticklebacks analysed from each bay.

Sampling was performed in May 2014, after adult sticklebacks had migrated from their offshore winter areas into the coastal zone to spawn. Sampling was conducted in 16 bays situated along a 350 km stretch of the central Swedish Baltic Sea coast (Fig 1). Shallow bays are important reproduction areas for many coastal fish species, including sticklebacks [19]. They are characterized by a diverse and highly productive community of aquatic vegetation and macro-invertebrates, many of which constitute potential prey for sticklebacks [20]. The 16 bays were selected to represent a mix of shallow bay habitats along an archipelago gradient from the mainland to the outer archipelago, including sheltered shallow lagoons with narrow inlets, to more open and exposed bays.

#### Stickleback sampling

Sampling of stickleback stomachs was performed as part of a larger survey targeting the whole food webs of shallow vegetated bays (see [11]). Sticklebacks were caught using Nordic survey gillnets (European Union 112 standardized method EN 14757:2005). The nets were set at 0.5–3 m depth between 4–7 pm, and lifted between 7–9 am the following morning. The fish died in the process of lifting the nets; after sticklebacks were removed from the nets they were immediately put in 95% ethanol. In total, 196 individual fish were analysed (Fig 1). In bays where fewer than 15 sticklebacks were caught, all of the fish were analysed with respect to their diet composition. For bays where more individuals were caught, a subset representing the size distribution in the catch was selected for the diet analyses.

The total length (TL) of each fish was measured to the nearest 1 mm. The mean total length was 57.7 ±7.6 (SD), with a range of 35–72 mm (S1 Fig). Visual inspection of the resulting size frequency distribution indicates a left skew, i.e. an underrepresentation of large individuals (S1 Fig), which was not an effect of skewed subsampling. Only 2.5% (5 of the 196 individuals) were >70 mm; a much smaller proportion than that found in other, similar surveys in the Western Baltic Sea (unpublished; [5]).

### Visual analysis of stomach content

Out of the 196 sticklebacks sampled, 192 were analysed using both visual methods and metabarcoding, and four were used in a pilot study for DNA metabarcoding. The stomachs were dissected and flushed with 80% EtOH to remove all stomach contents. To avoid cross-contamination, the dissection tools were rinsed with soap, bleach, and Milli-Q water before each individual dissection. Prey items visually distinguishable in the flushed stomach contents were identified to the highest taxonomic resolution possible, using a stereo microscope (magnification 20-80x). Frequency of occurrence for each prey item was estimated (%F_vis_, the percentage of stomachs in which a prey was present). Thereafter, all stomach contents were stored at -20°C in 80% EtOH for subsequent DNA extraction. The level of digestion for each stomach was classified on a 1–5 scale, where 1 = intact prey, 2 = partially digested, 3 = extensively digested, 4 = very few prey parts discernible, and 5 = fully digested/ empty stomach.

### DNA metabarcoding analysis

#### Sample processing

DNA was extracted from the 196 sticklebacks’ gut contents using the UltraClean® Tissue and Cells DNA Isolation Kit (MO BIO Laboratories), according to the manufacturer’s instructions. The dual PCR amplification method was used for Illumina MiSeq library preparation [21]. The cytochrome oxidase 1 (CO1) marker was first amplified using locus specific primers including an Illumina adapter overhang (amplicon PCR). The primers were based on Leray et al.’s (2013) [22] ‘mini-barcode’ yielding a 313 bp fragment (CO1mini_mICOIintF_MiSeq: TCGTCGGCAGCGTCAGATGTGTATAAGAGACAGGGWACWGGWTGAACWGTWTAYCCYCC, CO1_dgHCO2198_MiSeq: GTCTCGTGGGCTCGGAGATGTGTATAAGAGACAGTAAACTTCAGGGTGACCAAARAAYCA, CO1 specific sequence is shown in bold, and illumina adapter in regular font). A blocking primer was used in the amplicon PCR, to prevent amplification from *G*. *aculeatus*, following [23]. A Spacer C3 CPG was added to the 3’ end of the blocking primer to prevent elongation without affecting annealing properties, minimizing predator DNA amplification (G_aculeatus_block_Hco_2198: CAAAGAATCAAAATAAGTGTTGGTAAAGA-C3). For each sample, two independent PCR reactions were performed and later pooled, ensuring greater coverage of prey items amplified. In a second PCR step, Illumina dual index adapters were incorporated to the amplicons using a limited number of cycles (Index PCR).

Amplicon PCRs were performed as 30 μl reactions with 20pm of each primer and 100pm of blocking primer and using Pfu proofreading DNA polymerase (Promega). Cycling conditions were as follows: 2 min at 95°C (1x); 1 min at 95°C, 45s at 55°C, 1 min at 72°C (40x); 5 min at 72°C (1x); hold at 4°C. Amplicons were checked on a 2% agarose gel. Agencourt® AMPure® XP paramagnetic beads (Beckman Coulter) were then used to purify the PCR products [21]. For index PCR, the Illumina Nextera XT kit (96 indices, 384 samples) was used according to manufacturer’s instructions. Index PCR was performed as 50 μl reactions using 5 μl of cleaned up amplicons. Cycling conditions were as follows: 3 min at 95°C (1x); 30s at 95°C, 30s at 55°C, 30s at 72°C (8x); 5 min at 72°C (1x); hold at 4°C. Agencourt® AMPure® XP paramagnetic beads (Beckman Coulter) were then used to purify the PCR products, using a ratio of 0.8 that allows the selection of fragments larger than 200 bp. DNA quantification was carried out using a Qubit Fluorometer (Invitrogen) and the average fragment size was verified using Tapestation (Agilent Technologies). Pooled libraries were then sequenced as paired-ends using Illumina MiSeq Reagent v3, producing 30 103 790 paired-end reads of 300 bp in length.

#### Bioinformatic data processing and analysis

The processing steps were performed using Qiime (Quantitative Insights into Microbial Ecology) version 1.9.1 [24] and custom python scripts. Paired-end joining was done using the Qiime fastq-join tool. A 48% sequence loss was observed after the paired-end joining step due to poor sequence quality at read ends (the raw data are available from the NCBI sequence read archive under accession number SRP101702, BioProject number PRJNA378633). Dual indexes and Illumina overhangs were removed by the sequencing platform. Primer sequences were removed using a custom python script (https://github.com/Quiterie90/Primer_Removal), corresponding to a 30% loss (Table 1). Due to its stringency, the script quality filters sequences by removing incomplete reads or chimeras. Additional quality filtering with Qiime removed 3% of the reads. Finally, remaining chimeric reads were excluded using UCHIME [25], producing a final dataset (0.5% loss).

**Table 1:** Number of reads after each bioinformatic data processing step.

Paired-end joining	Primer trimming	Quality filtering
15 706 724	10 982 728	10 586 546

The Bayesian clustering algorithm CROP was used to cluster the sequences into operational taxonomic units (OTUs) based on the natural distribution of the data, using a Gaussian model [26]. According to a benchmarking study by Leray et al. [22], the best lower and upper bound values to cluster metazoan CO1 sequences are 3 and 4, corresponding to sequence dissimilarities between 6% and 8% (CROP -i <input.fasta> -b 211731 -z 470 -l 3 -u 4 -o <output>).

For taxonomic assignment of CO1 sequences, a custom database was created, consisting in a taxonomy file associated with a reference sequence file, of Metazoan sequences retrieved from BOLD (http://www.boldsystems.org/ downloaded in March 2016), combined with own reference databases of Chironomidae, Nemertea, Xenacoelomorpha and Oligochaeta and barcodes of Swedish Echinodermata, Mollusca, Cnidaria and Arthropoda from the Swedish Barcode of Life database (SweBol).

Taxonomic assignment was done using a 97% similarity threshold using the Uclust software implemented in Qiime with the default parameters [27]. In order to obtain matches for non-Metazoan taxa, we also did a Megablast search with a 97% similarity threshold, a minimum query coverage of 70% and an e-value inferior to 1E-100 against the Genbank nt (nucleotide) database (ftp://ftp.ncbi.nlm.nih.gov/blast/db/) with Geneious [28].

#### Data analysis

After sequencing, we obtained an OTU table showing the number of reads per taxon found in the stomach of each fish. For diet derived from this barcoding identification, frequency of occurrence was estimated (%F_bar_)—the percentage of stomachs in which a prey (OTU) was present.

To investigate the effect of fish body size (mm TL) on their diet and account for the hierarchical data structure, we performed permutational multivariate analysis of variance (PERMANOVA, *adonis* function in the vegan package for R [29] on the Bray–Curtis distance matrix with ‘bay’ (16 levels) as strata, fish size group as fixed predictor, and diet composition (counts of stomach with a certain prey present) as a response. Fish were divided into two size groups (TL): ≤6.5 cm (S), and >6.5 cm (L).

### Comparison of visual vs DNA-based methods

The results from the visual analysis and the metabarcoding analysis were compared with respect to both number of taxa identified and to the taxonomic resolution of the data. The number of taxa was the mean number of taxa identified per stickleback in the two methods applied. To compare the methods with respect to their taxonomic resolution, ranks were given to each prey item in each stomach and then mean taxonomic rank of the stomach was used [30]. Taxonomic resolution was ranked as follows: species = 1, genus = 2, family = 3, infra-order = 4, order = 5, infra-class = 6, class = 7, phylum = 8. Infra-class and infra-order represent taxonomic rankings between class and order and between family and order, respectively. Paired t-tests were used to compare the resolution between the methods.

## Results

### Diet composition based on DNA barcoding

**Fig 2:**
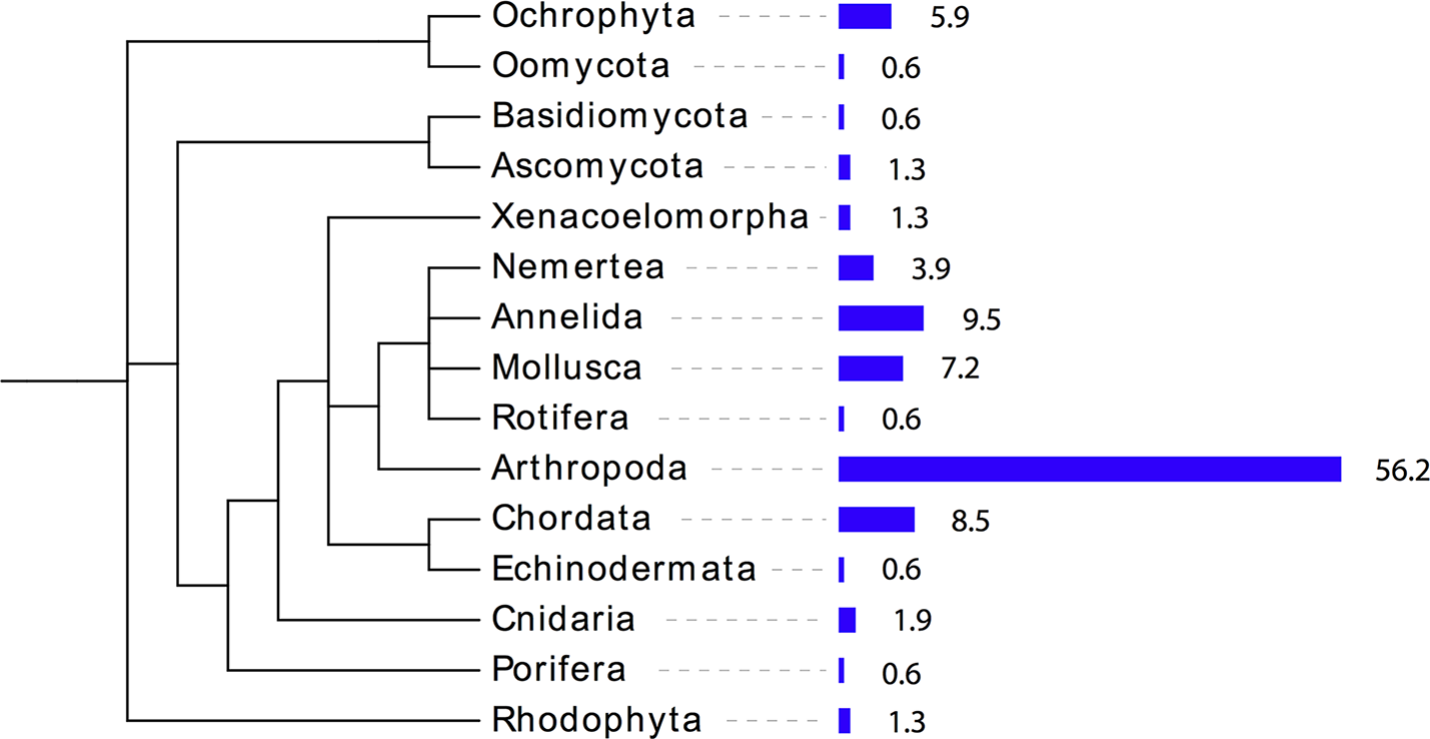
Frequencies of phyla identified in three-spined stickleback gut contents. Bar length corresponds to the frequency of OTU assigned to a specific phylum.

Using metabarcoding, 120 taxa were identified in the stomachs of sticklebacks: 15 phyla, 27 classes, 52 orders, 66 families, 83 genera, and 84 species (S1 Table). A broad range of phyla were found, but Arthropoda dominated by far (Fig 2). Given that this is the first barcoding-based study of Baltic Sea stickleback diet, we provide the whole list of taxa found (S1 Table). We only omit records from primates and birds, which were obviously contamination. Taxa likely to be accidental or secondary prey were also excluded from further analyses. Specifically, we excluded Fungi, Macroalgae and Chromista (as these are not targeted as food by sticklebacks), and kept only Metazoa in the primary prey list. A few OTUs of Metazoa were also excluded as they were either unlikely to be prey, or due to possible contamination (see S1 Table). In total, 103 taxa were considered primary prey and were used in the subsequent analyses.

Sticklebacks had a broad spectrum of prey items, of which Insecta (mainly chironomids), Maxillipoda (harpacticoid copepods) and Branchiopoda (cladocerans) were the dominating food items, found in more than 90% of the samples (S1 Table). At the species level, the main prey were the chironomid *Tanytarsus usmaensis*, the harpacticoid *Tachidius discipes*, and the cladoceran *Pleopis polyphemoides* (S1 Table).

**Fig 3:**
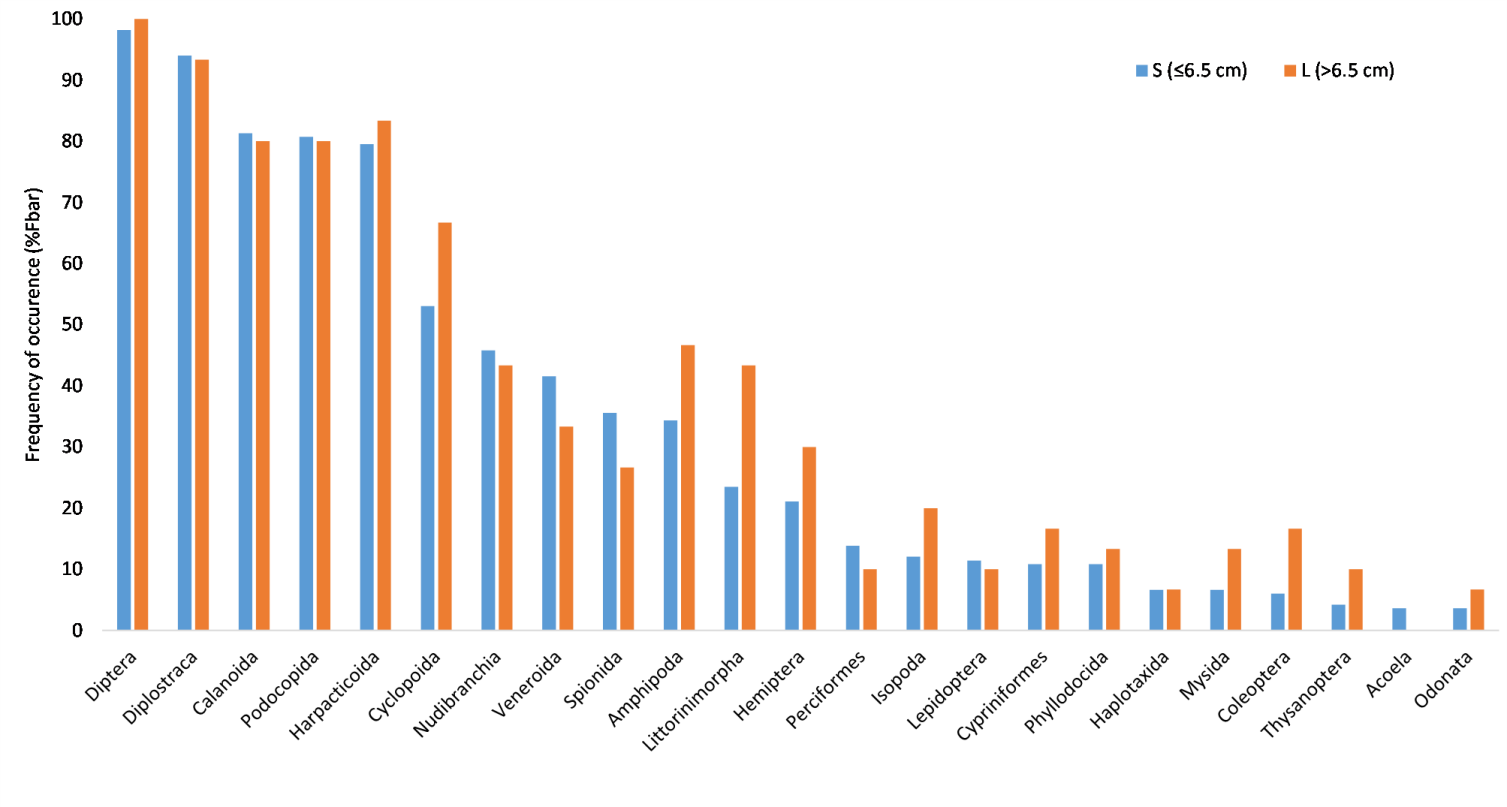
Diet composition of different size groups of stickleback (at order level). Only orders with >5% of frequency of occurrence are shown.

Although the range of stickleback body lengths was too small to detect ontogenetic diet shifts, significant differences in stomach content depending on fish size were found (PERMANOVA, F = 3.7, p = 0.01). The diet of the large fish (>6.5 cm) differed from the group of smaller fish (≤6.5 cm). Specifically, amphipods, isopods and gastropods appeared to be more common in the diet of the larger fish, as well as insects like hemipterans and coleopterans (Fig 3).

### Methods comparison: Visual identification vs DNA barcoding

**Fig 4:**
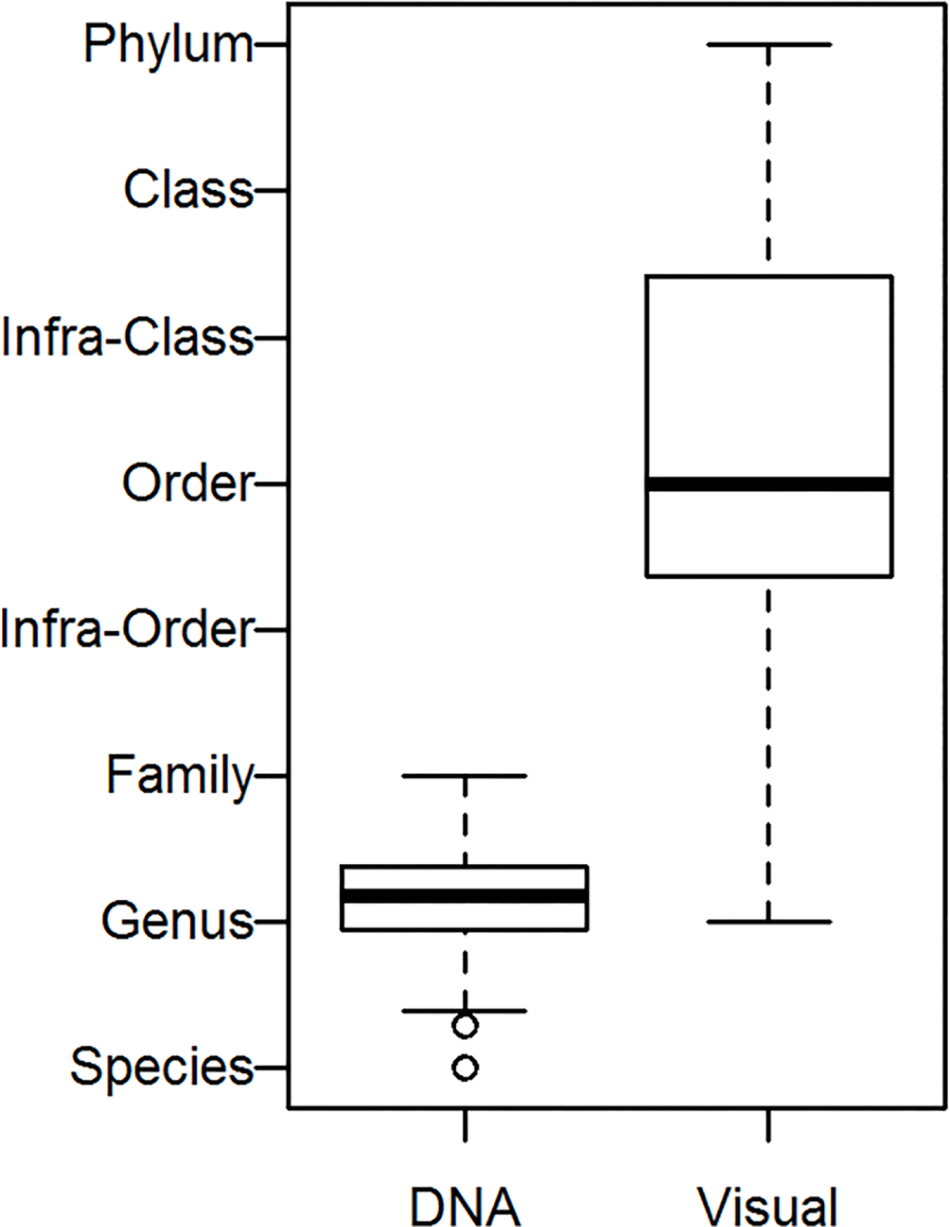
Mean taxonomic rank assigned to items within individual stomachs. DNA–assigned by barcoding, Visual–visual identification disregarding non-identified items. Midline represents median, boxes first and third quartiles, whiskers either maximum values or 1.5 times interquartile range (whichever is smaller) and circles outliers.

The taxonomic resolution of the prey identified differed substantially between the two methods. DNA barcoding gave a much higher resolution (with median rank of genus, p<0.0001). Disregarding stomachs for which no visual identification could be done, the median taxonomic rank for visual inspection was order (Fig 4).

DNA barcoding also resulted in a much higher number of prey taxa identified per stomach than visual analysis (p<0.0001): 21.7±8.8 vs 1.96±1 (mean±SD). Also the total number of taxa identified using DNA barcodes was much larger than the number of taxa identified using visual quantification (120 vs 21; see S1 and S2 Tables). The average level of digestion was 3.6, meaning that gut contents were extensively digested and/or with very few prey items present. Not surprisingly, many taxa identified using DNA barcodes could not have been identified visually (e.g. due to their small size). However, some taxa identified by visual inspection were not revealed by barcoding (*Temora longicornis*, *Bosminidae*, *Hydracarina*).

**Fig 5:**
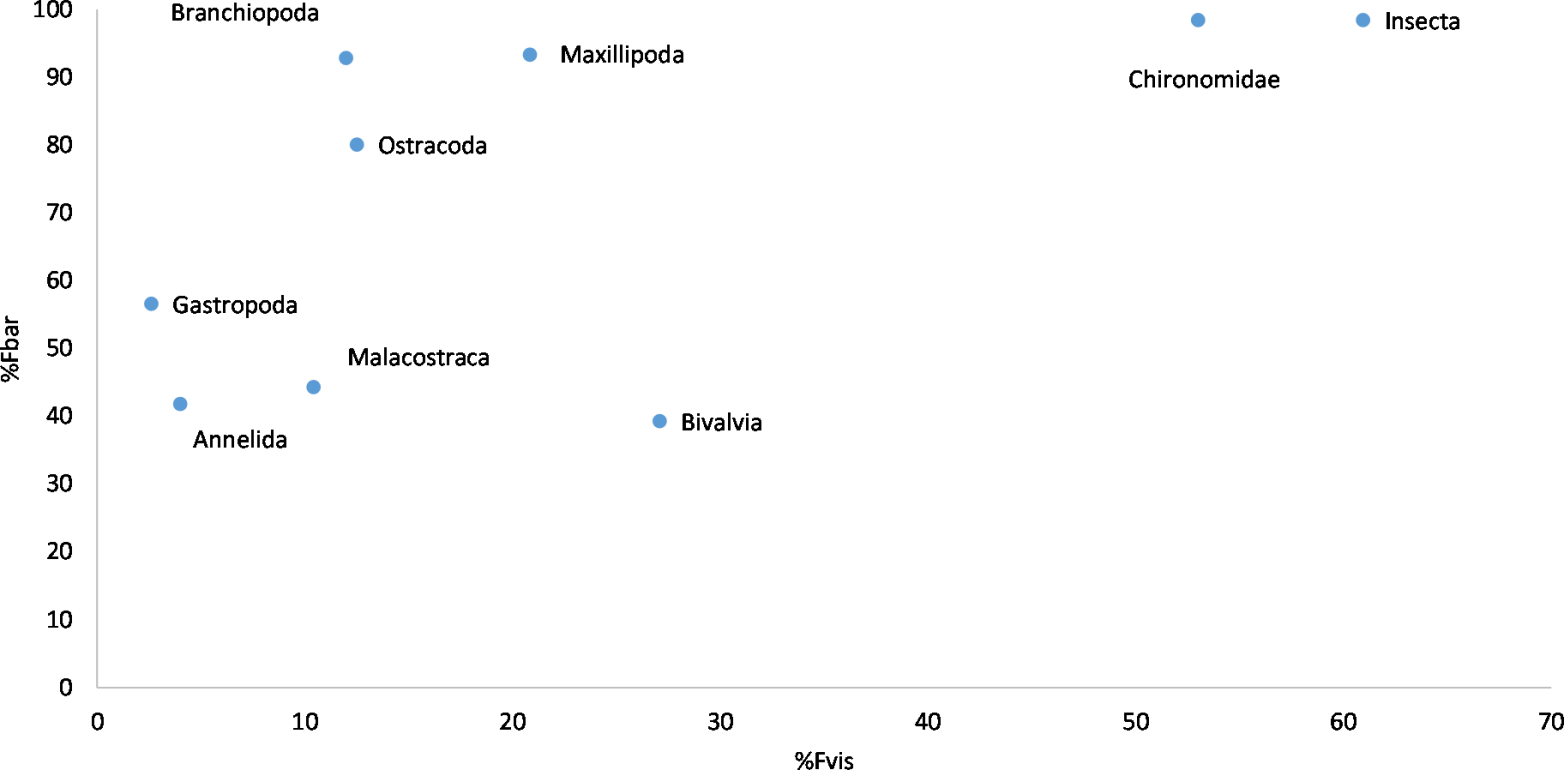
Diet of three-spined stickleback. Relationship between the results of two methods used: frequency of occurrence determined by metabarcoding (%F_bar_) and by visual analysis (%F_vis_).

Irrespective of these minor differences, the two methods showed consistent patterns: at the population level, frequency of occurrence determined by visual analysis (%F_vis_) corresponded well with the frequencies based on OTU reads (%F_bar_; Fig 5), although the relationship was not linear. Instead, a curvilinear relationship was seen, where the frequencies of occurrence in the metabarcoding analyses were higher than in the visual identification for all taxa. Such a relationship is to be expected, since barcoding is capable of detecting even very little amounts of the prey, which could not be detected visually. Only Bivalvia appeared to have very similar frequencies detected by both methods.

## Discussion

The aim of this study was to assess the diet of the three-spined stickleback in a coastal ecosystem, and to compare classic visual analysis and novel DNA metabarcoding for identifying fish prey in stomach contents. The main prey items found were chironomids, cladocerans and harpacticoid copepods. Large (>6.5 cm) sticklebacks had higher proportions of benthic herbivores, like amphipods, gastropods and isopods, in their diet. The results of the DNA barcoding revealed a highly diverse stickleback diet (more than 100 taxa in total, and >20 per individual) and provided a much higher taxonomic resolution than the conventional visual stomach content analysis.

### Diet composition

While stickleback diets are well studied from other parts of the world, no previous studies have revealed such a high diversity of prey items, most likely because of limitations in the methods used (for some examples of previous studies, see S3 Table). Sticklebacks, however, inhabit many different habitats and ecosystems, so their diet varies accordingly. In pelagic areas of the Baltic Sea, where sticklebacks spend a large part of their life, they feed primarily on cladocerans and calanoid copepods [31–33], but at the coast the main prey items are insect larvae, harpacticoids and amphipods [34,35]. In freshwater systems, they are known to prey on both planktonic and benthic prey. We found chironomids and harpacticoids to be a very important part of the stickleback diet, similar to the diet in the coastal zone of the Bothnian Bay, in the northern Baltic Sea [35].

From a food-web perspective, the high abundance of cladocerans found in the diet (Fig 3, S1 Table) might indicate competition with juvenile stages of other fish, especially when preference for cladocerans is evident [33,36,37]. Ljunggren et al. (2010) [6] suggested that recruitment of coastal predatory fish in the Baltic Sea (pike and perch) was impaired by limited food availability (zooplankton) for their larvae, due to competition with sticklebacks. The three-spined stickleback has indeed been shown to deplete zooplankton communities in brackish water lagoons with similar densities as in the current study area [38]. On the other hand, sticklebacks have also been shown to feed on small pike and perch larvae, which would constitute a more direct effect on populations of large predators, than competition [12]. We detected *Perciformes* in the stomachs of six fish (see S1 Table), potentially indicating sticklebacks may have been feeding on perch egg or larvae.

Concerning benthic prey, the most significant part of the diet consisted of chironomid larvae, which were one of the most common epibenthic organism groups in the 16 bays. Sticklebacks are well-known to feed on chironomids in freshwater areas ([35,36,39,40], S3 Table). Chironomids are a broad taxonomic group, with a diverse diet spanning between phytoplankton, epiphytic algae, detritus, macrophytes, and crustacean zooplankton [41]. More knowledge on the role of chironomids in the food webs and the interactions with sticklebacks is needed, since possible cascading effects from sticklebacks via chironomids to lower trophic levels may be present (e.g., [42]).

Sticklebacks seemed to have fed less on the gammarid amphipods than expected from previous experimental studies, where they have been shown to strongly reduce gammarid densities in the lab and in the field [7,9]. In our study, larger sticklebacks appear to feed more on amphipods (Fig 3). It is well known that stickleback mouth width and gape size influence the size of prey that can be eaten [43,44], and that jaw morphology (gape size, gill raker spacing) can change food handling efficiency [45]. Therefore, the optimum diet might differ between stickleback populations and/or habitats depending on their morphology. Hart and Ison (1991) found the size threshold of prey rejection to be at 6–7 mm [44], Byström et al. (2015) suggests the upper limit to be around 5 mm [12]. Given that fish above 5 cm eat amphipods [34,46], there were plenty of gammarids of appropriate size for sticklebacks to eat (see S2 Fig), showing that mouth morphology does not explain rejection of amphipods in small stickleback.

The most likely explanation for the relatively low proportion of gammarids in the overall diet is an underrepresentation of large individuals sampled in this study (S1 Fig) compared to several previous studies ([5]; and unpublished). These large individuals appear to feed the most on gammarids (Fig 3). The underrepresentation in the nets, which were placed at > 1m depth, may indicate that the largest sticklebacks occupy the most beneficial habitats in the bays, i.e. the shallowest vegetated parts, where we could not fish using gillnets. These shallow areas are also the habitats with the highest abundances of gammarids, which may have led to the low frequency of stickleback predation upon gammarids apparent in the analysis. Large sticklebacks (>6.5 cm) also tend to have a higher frequency of occurrences of cyclopoid copepods than smaller ones (Fig 3), mainly driven by *Eucyclops macruroides*. This species is known to inhabit vegetation in the littoral zone, which again supports a possible small-scale differences in foraging habitats between stickleback size classes.

In many of the 16 bays there were relatively few stickleback individuals sampled, resulting in the inability to assess individual specialisation. To assess the link between sticklebacks and large, benthic crustaceans (e.g., gammarids), more detailed and intense sampling should be conducted, and the potential for individual specialisation should be investigated.

### Visual inspection vs DNA barcoding

In general, the two methods gave consistent results with the same prey taxa dominating (Fig 5). However, as the stomach content was extensively digested and/or with very few prey items present, the visual prey species identification was in many cases obscured. Fish may have been caught in the nets up to 12 hours before they were preserved, making the visual analysis particularly difficult. On the other hand, this may also have had an effect on DNA degradation. In diet studies, a high proportion of unidentifiable material in the guts, which cannot be visually assigned to any prey category, is common [15]. Even though both methods are time-consuming and expensive, and despite the fact that some prey species were missed (*Temora longicornis*, *Bosminidae*, Hydracarina), barcoding provided a much higher taxonomic resolution and therefore produced a more accurate and detailed analysis of gut contents In terms of the resolution provided by the two methods, our results are similar to a previous fish feeding ecology study [30].Thus, we consider these discrepancies as minor, since barcoding still enabled the disclosure of unexpectedly high diversity in the stickleback diet.

### Methodological shortcomings

The results of DNA metabarcoding and the visual analysis did not match fully.—Some prey taxa (*Temora longicornis*, *Bosminidae*, Hydracarina) were detected by visual inspection only, while Bivalvia had very similar frequency values estimated by both methods (see Fig 5). As we could visually identify these prey organisms, their DNA is unlikely to have been too degraded for barcoding to identify them. A more likely explanation is that even though the CO1 primers are designed to be taxonomically broad they may not bind equally well to all prey species, and maybe not at all to some. It is known that even minor primer–template mismatches can lead to substantial under-representation of the prey in the diet [47]. These biases are then accumulated through DNA amplifications during the PCR reaction [48,49]. Bosminidae was identified during visual inspection of stomach contents, but when barcoded only a higher corresponding taxon was detected (Diplostraca). Thus, only species or group specific primers would guarantee the most accurate identification.

Blocking primers are used to avoid ‘predator sequences’ (i.e. lots of non-informative reads), which can reduce potential of prey detection [50], but could also block prey DNA [51], which may bring in bias when analysing mixtures of DNA. We used a blocking primer to avoid stickleback sequences, but since predator and prey missed are not phylogenetically close, and the blocking primer used is specific to *G*. *aculeatus*, this should not have impaired the results.

There can be other biases introduced during the bioinformatic analysis steps, such as during the clustering of sequences, where the number of OTUs or ‘species’ found depends on the sequence similarity cut-off used, and during taxonomic assignment, which uses a sequence identity threshold of 97%. Also, it is obvious that if some species are not represented in the DNA reference library, no matches for these will be found.

Secondary consumption, i.e., prey of the prey, parasites or accidental material consumed during feeding, may confound the results in DNA-based studies [52–54]. The magnitude of potential error due to secondary predation depends on digestion rates [54]. We acknowledge that even though a few unlikely prey taxa were removed from the analysis, some secondary prey may still have been included in the analysis as primary prey items. However, DNA of secondary prey might be expected to represent only a minor part of total OTU reads compared to primary prey, due to a much lower total biomass and to a higher level of degradation.

When visually inspecting the often highly degraded stomach content, prey items such as fish eggs and larvae may be substantially underestimated (e.g., [55]). Although metabarcoding has the power to catch such prey species, the life stages of prey items remain unknown. Moreover, prey analyses based on stomach content only represent a snapshot in time. To obtain more comprehensive knowledge on stickleback diets, future studies should be complemented with analyses of stable isotopes/fatty acids, which integrate the signal from different prey organisms over longer time.

Despite these shortcomings, DNA metabarcoding seems to be a viable method to assess stickleback diets. From a data quality perspective, we therefore, at least until metabarcoding methods are further developed, suggest to combine high-throughput DNA sequencing and traditional visual stomach content analysis, to achieve the best resolution of diet composition and diversity.

### Implications

Using a powerful combination of visual and metabarcoding-based analyses of stomach contents, we show that the three-spined stickleback feeds on a wide variety of organisms in coastal areas of the Baltic Sea, including pelagic zooplankton and benthic epifaunal invertebrates. As a consequence, the major increase in stickleback abundance [5] could affect many parts of both pelagic and benthic food webs, resulting in competition with other fish species, and cascading effects down to primary producers [7,11]. Given that the expected increase in the Baltic Sea surface water temperatures [56] may be beneficial for stickleback population growth [57], studies such as this one could provide important information about the current and future impacts of three-spined sticklebacks on the Baltic Sea ecosystem.

## Supporting information

S1 Table Taxa found in three-spined stickleback stomachs as revealed by DNA metabarcoding (Primates and Aves excluded). Items in italics were considered as secondary/ accidental prey %F_bar_- frequency of occurrence (percentage of stomachs in which a prey was present).
(DOCX)

S2 Table Diet of three-spined stickleback as revealed by visual stomach content analysis. %F_vis_—the percentage of stomachs in which a prey was present.
(DOCX)

S3 Table Summary of some studies on three-spined stickleback diet. (DOCX)

S1 Fig Stickleback size (total length, mm) distribution in a sample. (TIF)

S2 Fig Gammaridae size distribution in the bays studied. (TIF)

S1 Appendix Comparison of quantification from OTU reads and results of visual stomach content analysis. (DOCX)
