# Supplementary material for: DNA metabarcoding reveals diverse diet of the three-spined stickleback in a coastal ecosystem
Source: PLoS One. 2017 Oct 23;12(10):e0186929. doi: 10.1371/journal.pone.0186929 (PMC5653352; doi:10.1371/journal.pone.0186929)
Supplement: S1 Table — Items in italics were considered as secondary/ accidental prey %Fbar- frequency of occurrence (percentage of stomachs in which a prey was present). (DOCX) [file pone.0186929.s001.docx]

**S1 Table**. **Taxa found in three-spined stickleback stomachs as revealed by DNA metabarcoding** (Primates and Aves excluded). Items in italics were considered as secondary/ accidental prey. %F_bar_- frequency of occurrence (percentage of stomachs in which a prey was present).

| Phylum | Class | Order | Family | Genus | Species | %F_bar_ |
| --- | --- | --- | --- | --- | --- | --- |
| Annelida | Clitellata | Haplotaxida | Tubificidae | Paranais | Paranais frici | 1,02 |
| Annelida | Clitellata | Haplotaxida | Tubificidae | Tubificoides | Tubificoides benedii | 0,51 |
| Annelida | Clitellata | Haplotaxida |  |  |  | 5,10 |
| Annelida | Polychaeta | Phyllodocida | Nereididae | Hediste | Hediste diversicolor | 8,67 |
| Annelida | Polychaeta | Phyllodocida | Polynoidae | Polynoidae sp. |  | 1,02 |
| Annelida | Polychaeta | Phyllodocida | Sigalionidae | Pisione | Pisione remota | 0,51 |
| Annelida | Polychaeta | Phyllodocida |  |  |  | 0,51 |
| Annelida | Polychaeta | Spionida | Spionidae | Marenzelleria | Marenzelleria arctia | 3,57 |
| Annelida | Polychaeta | Spionida | Spionidae | Marenzelleria | Marenzelleria viridis | 32,65 |
| Annelida | Polychaeta | Spionida |  |  |  | 2,55 |
| Annelida | Polychaeta | Terebellida | Ampharetidae | Mugga | Mugga wahrbergi | 0,51 |
| Annelida | Polychaeta | Terebellida | Cirratulidae | Chaetozone | Chaetozone setosa | 0,51 |
| **Annelida total** |  |  |  |  |  | 41,84 |
| Arthropoda | Branchiopoda | Diplostraca | Daphniidae | Daphnia | Daphnia cucullata | 0,51 |
| Arthropoda | Branchiopoda | Diplostraca | Daphniidae | Daphnia |  | 2,04 |
| Arthropoda | Branchiopoda | Diplostraca | Leptodoridae | Leptodora | Leptodora kindtii | 0,51 |
| Arthropoda | Branchiopoda | Diplostraca | Macrothricidae | Macrothrix |  | 0,51 |
| Arthropoda | Branchiopoda | Diplostraca | Podonidae | Evadne | Evadne nordmanni | 59,69 |
| Arthropoda | Branchiopoda | Diplostraca | Podonidae | Pleopis | Pleopis polyphemoides | 87,76 |
| Arthropoda | Branchiopoda | Diplostraca | Sididae | Sida | Sida crystallina | 14,80 |
| Arthropoda | Branchiopoda | Diplostraca |  |  |  | 34,18 |
| Arthropoda | **Branchiopoda total** |  |  |  |  | 92,86 |
| Arthropoda | Collembola | Entomobryomorpha | Isotomidae | Isotoma | Isotoma anglicana | 0,51 |
| Arthropoda | Collembola | Symphypleona | Sminthuridae | Allacma | Allacma fusca | 0,51 |
| *Arthropoda* | *Insecta* | *Coleoptera* | *Curculionidae* | *Polydrusus* | *Polydrusus cervinus* | *1,02* |
| Arthropoda | Insecta | Coleoptera | Scirtidae | Cyphon | Cyphon padi | 7,65 |
| Arthropoda | Insecta | Diptera | Chironomidae | Chironomus | Chironomus aprilinus | 78,06 |
| Arthropoda | Insecta | Diptera | Chironomidae | Chironomus | Chironomus plumosus | 73,47 |
| Arthropoda | Insecta | Diptera | Chironomidae | Cladotanytarsus | Cladotanytarsus pallidus | 58,67 |
| Arthropoda | Insecta | Diptera | Chironomidae | Cricotopus | Cricotopus bicinctus | 63,27 |
| Arthropoda | Insecta | Diptera | Chironomidae | Cricotopus | Cricotopus patens | 30,61 |
| Arthropoda | Insecta | Diptera | Chironomidae | Cryptochironomus | Cryptochironomus supplicans | 3,57 |
| Arthropoda | Insecta | Diptera | Chironomidae | Dicrotendipes | Dicrotendipes modestus | 66,33 |
| Arthropoda | Insecta | Diptera | Chironomidae | Dicrotendipes | Dicrotendipes nervosus | 5,61 |
| Arthropoda | Insecta | Diptera | Chironomidae | Dicrotendipes | Dicrotendipes tritomus | 7,65 |
| Arthropoda | Insecta | Diptera | Chironomidae | Orthocladius | Orthocladius oblidens | 77,04 |
| Arthropoda | Insecta | Diptera | Chironomidae | Procladius | Procladius culiciformis | 35,20 |
| Arthropoda | Insecta | Diptera | Chironomidae | Psectrocladius |  | 34,69 |
| Arthropoda | Insecta | Diptera | Chironomidae | Tanytarsus | Tanytarsus cf. longitarsis | 43,88 |
| Arthropoda | Insecta | Diptera | Chironomidae | Tanytarsus | Tanytarsus mendax | 3,06 |
| Arthropoda | Insecta | Diptera | Chironomidae | Tanytarsus | Tanytarsus usmaensis | 96,94 |
| Arthropoda | Insecta | Diptera | **Chironomidae total** |  |  | 98,47 |
| Arthropoda | Insecta | Diptera |  |  |  | 79,59 |
| Arthropoda | Insecta | Hemiptera |  |  |  | 21,94 |
| Arthropoda | Insecta | Lepidoptera |  |  |  | 11,22 |
| Arthropoda | Insecta | Odonata | Libellulidae | Sympetrum |  | 0,51 |
| Arthropoda | Insecta | Odonata |  |  |  | 3,57 |
| Arthropoda | Insecta | Thysanoptera |  |  |  | 5,10 |
| Arthropoda | Insecta | Trichoptera |  |  |  | 1,53 |
| Arthropoda | Insecta |  |  |  |  | 3,06 |
| Arthropoda | **Insecta total** |  |  |  |  | 98,47 |
| Arthropoda | Malacostraca | Amphipoda | Gammaridae | Gammarus | Gammarus tigrinus | 16,84 |
| Arthropoda | Malacostraca | Amphipoda | Pontoporeiidae | Monoporeia | Monoporeia affinis | 0,51 |
| Arthropoda | Malacostraca | Amphipoda |  |  |  | 32,14 |
| Arthropoda | Malacostraca | Isopoda | Asellidae | Asellus | Asellus aquaticus | 12,24 |
| Arthropoda | Malacostraca | Isopoda | Janiridae | Jaera | Jaera albifrons | 1,53 |
| Arthropoda | Malacostraca | Mysida | Mysidae | Neomysis | Neomysis integer | 5,61 |
| Arthropoda | Malacostraca | Mysida | Mysidae | Praunus | Praunus inermis | 3,06 |
| Arthropoda | **Malacostraca total** |  |  |  |  | 44,39 |
| Arthropoda | Maxillopoda | Calanoida | Acartiidae | Acartia | Acartia bifilosa | 36,73 |
| Arthropoda | Maxillopoda | Calanoida | Acartiidae | Acartia | Acartia tonsa | 33,16 |
| Arthropoda | Maxillopoda | Calanoida | Centropagidae | Limnocalanus | Limnocalanus macrurus | 0,51 |
| Arthropoda | Maxillopoda | Calanoida | Diaptomidae | Eudiaptomus | Eudiaptomus graciloides | 4,08 |
| Arthropoda | Maxillopoda | Calanoida | Temoridae | Eurytemora | Eurytemora affinis | 75,51 |
| Arthropoda | Maxillopoda | Calanoida |  |  |  | 59,18 |
| Arthropoda | Maxillopoda | Cyclopoida | Cyclopidae | Cyclops | Cyclops abyssorum | 4,08 |
| Arthropoda | Maxillopoda | Cyclopoida | Cyclopidae | Eucyclops | Eucyclops cf. serrulatus | 13,27 |
| Arthropoda | Maxillopoda | Cyclopoida | Cyclopidae | Eucyclops | Eucyclops macruroides | 13,27 |
| Arthropoda | Maxillopoda | Cyclopoida | Cyclopidae | Macrocyclops | Macrocyclops distinctus | 3,57 |
| Arthropoda | Maxillopoda | Cyclopoida | Cyclopidae |  |  | 47,96 |
| Arthropoda | Maxillopoda | Harpacticoida | Tachidiidae | Tachidius | Tachidius discipes | 79,59 |
| Arthropoda | Maxillopoda | Sessilia | Balanidae | Amphibalanus | Amphibalanus improvisus | 1,53 |
| Arthropoda | **Maxillopoda total** |  |  |  |  | 93,37 |
| Arthropoda | Ostracoda | Podocopida | Cytherideidae | Cyprideis | Cyprideis torosa | 16,84 |
| Arthropoda | Ostracoda | Podocopida |  |  |  | 78,06 |
| Arthropoda | **Ostracoda total** |  |  |  |  | 80,10 |
| *Ascomycota* | *Eurotiomycetes* | *Eurotiales* | *Trichocomaceae* | *Penicillium* | *Penicillium digitatum* | *0,51* |
| *Ascomycota* | *Eurotiomycetes* | *Eurotiales* | *Trichocomaceae* | *Penicillium* | *Penicillium sclerotiorum* | *4,08* |
| *Basidiomycota* | *Microbotryomycetidae* | *Sporidiobolales* | *Sporidiobolaceae* | *Rhodotorula* | *Rhodotorula taiwanensis* | *0,51* |
| Chordata | Actinopterygii | Cypriniformes | Cyprinidae | Abramis | Abramis brama | 6,12 |
| Chordata | Actinopterygii | Cypriniformes | Cyprinidae | Phoxinus | Phoxinus phoxinus | 8,67 |
| Chordata | Actinopterygii | Cypriniformes | Cyprinidae | Tinca | Tinca tinca | 0,51 |
| Chordata | Actinopterygii | Cypriniformes |  |  |  | 3,06 |
| Chordata | Actinopterygii | Clupeiformes |  |  |  | 1,02 |
| Chordata | Actinopterygii | Gasterosteiformes | Gasterosteidae | Pungitius | Pungitius pungitius | 79,08 |
| Chordata | Actinopterygii | Perciformes | Gobiidae | Gobius | Gobius niger | 0,51 |
| Chordata | Actinopterygii | Perciformes | Gobiidae | Pomatoschistus | Pomatoschistus microps | 4,59 |
| Chordata | Actinopterygii | Perciformes | Gobiidae | Pomatoschistus | Pomatoschistus minutus | 7,14 |
| Chordata | Actinopterygii | Perciformes | Percidae | Gymnocephalus | Gymnocephalus cernua | 2,55 |
| Chordata | Actinopterygii | Perciformes |  |  |  | 3,06 |
| *Chordata* | *Actinopterygii* | *Salmoniformes* | *Salmonidae* | *Salmo* | *Salmo trutta* | *0,51* |
| Chordata | Leptocardii | Amphioxiformes | Branchiostomidae | Branchiostoma | Branchiostoma lanceolatum | 0,51 |
| Cnidaria | Hydrozoa | Anthoathecata | Hydridae | Hydra | Hydra oligactis | 1,02 |
| Cnidaria | Hydrozoa | Leptothecata | Melicertidae | Melicertum | Melicertum octocostatum | 0,51 |
| Cnidaria | Scyphozoa | Semaeostomeae | Ulmaridae | Aurelia | Aurelia aurita | 0,51 |
| Echinodermata | Ophiuroidea | Ophiurida |  |  |  | 2,04 |
| Mollusca | Bivalvia | Veneroida | Cardiidae | Cerastoderma | Cerastoderma glaucum | 10,71 |
| Mollusca | Bivalvia | Veneroida | Semelidae | Abra | Abra nitida | 0,51 |
| Mollusca | Bivalvia | Veneroida | Tellinidae | Macoma | Macoma balthica | 31,63 |
| Mollusca | **Bivalvia total** |  |  |  |  | 39,29 |
| Mollusca | Gastropoda | Hygrophila | Lymnaeidae | Radix |  | 0,51 |
| Mollusca | Gastropoda | Littorinimorpha | Hydrobiidae | Hydrobia | Hydrobia ulvae | 26,02 |
| Mollusca | Gastropoda | Littorinimorpha | Hydrobiidae | Potamopyrgus | Potamopyrgus antipodarum | 0,51 |
| Mollusca | Gastropoda | Mesogastropoda | Eulimidae | Haliella | Haliella stenostoma | 0,51 |
| Mollusca | Gastropoda | Nudibranchia | Calmidae | Calma | Calma glaucoides | 43,88 |
| Mollusca | Gastropoda | Nudibranchia |  |  |  | 3,06 |
| *Mollusca* | *Gastropoda* | *Stylommatophora* | *Agriolimacidae* | *Deroceras* | *Deroceras reticulatum* | *0,51* |
| Mollusca | **Gastropoda total** |  |  |  |  | 56,63 |
| Nemertea | Anopla |  | Lineidae | Lineus |  | 1,53 |
| Nemertea | Anopla |  |  |  |  | 1,53 |
| Nemertea | Enopla | Monostilifera | Emplectonematidae | Nemertopsis |  | 0,51 |
| Nemertea | Enopla | Monostilifera | Tetrastemmatidae | Tetrastemma |  | 1,53 |
| Nemertea | Enopla | Monostilifera |  |  |  | 0,51 |
| Nemertea | Enopla |  |  |  |  | 2,04 |
| *Ochrophyta* | *Bacillariophyceae* | *Cymbellales* | *Gomphonemataceae* | *Gomphonema* | *Gomphonema parvulum* | *0,51* |
| *Ochrophyta* | *Bacillariophyceae* | *Melosirales* | *Melosiraceae* | *Melosira* | *Melosira ambiqua* | *1,02* |
| *Ochrophyta* | *Bacillariophyceae* | *Melosirales* | *Melosiraceae* | *Melosira* | *Melosira nummuloides* | *16,84* |
| *Ochrophyta* | *Bacillariophyceae* | *Thalassiosirales* | *Skeletonemaceae* | *Skeletonema* | *Skeletonema marinoi* | *0,51* |
| *Ochrophyta* | *Eustigmatophyceae* | *Eustigmatales* | *Monodopsidaceae* | *Nannochloropsis* | *Nannochloropsis limnetica* | *9,18* |
| *Ochrophyta* | *Phaeophyceae* | *Ectocarpales* | *Acinetosporaceae* | *Pylaiella* | *Pylaiella washingtoniensis* | *19,39* |
| *Ochrophyta* | *Phaeophyceae* | *Ectocarpales* | *Chordariaceae* | *Leathesia* | *Leathesia difformis* | *1,02* |
| *Ochrophyta* | *Phaeophyceae* | *Ectocarpales* | *Ectocarpaceae* | *Ectocarpus* |  | *1,53* |
| *Ochrophyta* | *Phaeophyceae* | *Laminariales* | *Chordaceae* | *Chorda* | *Chorda filum* | *1,02* |
| *Oomycota* | *Peronosporea* | *Pythiales* | *Pythiaceae* | *Pythium* |  | *11,22* |
| Porifera | Demospongiae | Halichondrida |  |  |  | 0,51 |
| *Rhodophyta* | *Florideophyceae* | *Ceramiales* | *Callithamniaceae* | *Aglaothamnion* | *Aglaothamnion roseum* | *2,55* |
| Rotifera | Monogononta | Ploima | Brachionidae | Brachionus | Brachionus calyciflorus | 0,51 |
| Xenacoelomorpha |  | Acoela | Actinoposthiidae | Philactinoposthia | Philactinoposthia saliens | 3,06 |
| Xenacoelomorpha |  | Acoela | Isodiametridae | Aphanostoma |  | 0,51 |
| Total number of taxa identified | |  |  |  |  | **120** |
| Total number of diet items (secondary/accidental items excluded) | | |  |  |  | **103** |
