# Supplementary material for: DNA metabarcoding reveals diverse diet of the three-spined stickleback in a coastal ecosystem
Source: PLoS One. 2017 Oct 23;12(10):e0186929. doi: 10.1371/journal.pone.0186929 (PMC5653352; doi:10.1371/journal.pone.0186929)
Supplement: S2 Table — %Fvis—the percentage of stomachs in which a prey was present. (DOCX) [file pone.0186929.s002.docx]

**S2 Table**. **Diet of three-spined stickleback as revealed by visual stomach content analysis**. %F_vis_ - the percentage of stomachs in which a prey was present.

| Phylum | Class | Order | Family | Genus + species | Identification name | %F_vis_ |
| --- | --- | --- | --- | --- | --- | --- |
| Algae |  |  |  |  | Algae | 0.52 |
| Mollusca | Bivalvia |  |  |  | Bivalvia | 27.08 |
|  | Gastropoda |  |  |  | Gastropoda | 2.60 |
| Arthropoda | Arachnida | Hydracarina |  |  | Acari | 1.56 |
|  | Branchiopoda | Diplostraca | Bosminidae | Bosmina spp. | Bosmina | 7.29 |
|  |  |  | Chydoridae |  | Chydoridae | 1.56 |
|  |  |  | Podonidae | Podon sp. | Podon | 2.08 |
|  | Branchiopoda | Diplostraca |  |  | Cladocera | 3.13 |
|  | **Branchiopoda total** |  |  |  |  | 12 |
|  | Maxillopoda | Calanoida | Temoridae | Eurytemora affinis | Eurytemora affinis | 2.08 |
|  |  |  | Temoridae | Temora longiremis | Temora longiremis | 2.08 |
|  |  | Harpacticoida |  |  | Harpacticoida | 2.60 |
|  |  |  |  |  | Copepoda | 16.67 |
|  | **Maxillopoda total** |  |  |  |  | 20.83 |
|  | Insecta |  | Chironomidae |  | Chironomidae | 52.60 |
|  | Insecta |  |  |  | Insecta | 8.85 |
|  | **Insecta total** |  |  |  |  | 60.93 |
|  | Malacostraca | Amphipoda |  |  | Amphipoda | 6.25 |
|  |  | Mysida | Mysidae |  | Mysidae | 0.52 |
|  | Malacostraca |  |  |  | Malacostraca | 3.65 |
|  | **Malacostraca total** |  |  |  |  | 10.42 |
|  | Ostracoda |  |  |  | Ostracoda | 12.50 |
| Arthropoda |  |  |  |  | Eggs (Copepoda/Cladocera) | 20.31 |
| Annelida |  |  |  |  | Segmented worms | 3.65 |
| Nematoda/Nemertea |  |  |  |  | Other worms | 2.60 |
| Total number of diet items | |  |  |  |  | **21** |
