# Supplementary material for: DNA metabarcoding reveals diverse diet of the three-spined stickleback in a coastal ecosystem
Source: PLoS One. 2017 Oct 23;12(10):e0186929. doi: 10.1371/journal.pone.0186929 (PMC5653352; doi:10.1371/journal.pone.0186929)
Supplement: S3 Table — (DOCX) [file pone.0186929.s003.docx]

**S3 Table. Summary of some studies on three-spined stickleback diet.**

| Stickleback size (TL, mm) | Prey item  (in descending order of abundance) | Season | Method for identification | Location | Longitude and latitude | Salinity  Marine, brackish, freshwater | Habitat  Pelagic  Benthic | Reference |
| --- | --- | --- | --- | --- | --- | --- | --- | --- |
| 20 – 70 | Bosmina longispina,  Eurytemora affinis,  Cercopagis pengoi | Early autumn (September 2- 6) | Visual | Baltic Sea, Gulf of Finland | Many locations in Gulf of Finland | brackish | pelagic | Peltonen H, Vinni M, Lappalainen A, Ponni J. Spatial feeding patterns of herring (L.), sprat (L.), and the three-spined stickleback (L.) in the Gulf of Finland, Baltic Sea. ICES J Mar Sci. 2004;61: 966–971. doi:10.1016/j.icesjms.2004.06.008 |
| ~45 – 75 | Bosmina coregoni, Eurytemora affinis,  Podon polyphemoides,  Acartia bifilosa | Summer (August) | Visual | Baltic Sea,  Gulf of Bothnia | Whole Gulf of Bothnia (many locations) | brackish | pelagic | Leinikki J. The diet of three-spined stickleback in the Gulf of Bothnia during its open water phase. Aqua Fenn. 1995;25: 71–75. |
| NA | Diptera (Chironomidae, Culicidae), Crustacea (Harpacticoida)  Amphipoda (Gammarus)  Mysidacea,  Ostracoda | Early summer  (May-June) | Visual | Baltic Sea,  Bay of Bothnia | 63°30’N  22°20’E | brackish | coastal, exposed sandy beach, shallower<50 cm | Frande C, Kjellman J, Leskela A, Hudd R. The food of three-spined stickleback (Gasterosteus aculeatus) on a whitefish (Coregonus lavaretus) nursery area in the bay of Bothnia. Aqua Fenn. 1993; 85–87. |
| NA | Bosmina longispina,  E. affinis,  C. pengoi  Podon spp.  Acartia spp. | Summer (July) | Visual | Baltic Sea,  Gulf of Riga | Many locations | brackish | pelagic | Lankov A, Ojaveer H, Simm M, Põllupüü M, Möllmann C. Feeding ecology of pelagic fish species in the Gulf of Riga (Baltic Sea): the importance of changes in the zooplankton community. J Fish Biol. 2010;77: 2268–84. doi:10.1111/j.1095-8649.2010.02805.x |
| 33 - 70 | Eurytemora affinis, Temora longicornis,  Acartia spp.  Eurytemora affinis,  Bosmina spp.  Acartia spp.  Bosmina spp.  Acartia spp. | Spring  Summer  Autumn | Visual | Southwest Baltic Sea |  | brackish | pelagic | Jakubavičiūtė E, Casini M, Ložys L, Olsson J. Seasonal dynamics in the diet of pelagic fish species in the southwest Baltic Proper. ICES J Mar Sci J du Cons. 2017;74: 750–758. doi:10.1093/icesjms/fsw224 |
| Juvenile  9 – 27 | Copepods (Temora longicornis,  Microsetella norvegica)  Ciliophoran (Helicostomella subulata)  Oligochaetae  Orthocladiinae | August - September | Visual | White Sea,  Seldianaya Inlet of Kandalaksha Bay | 66°20′14.5′′N 33°37′27.8′′E  66°20′N 33°37′E | marine | coastal Zostera seagrass beds | Demchuk A, Ivanov M, Ivanova T, Polyakova N, Mas-Martí E, Lajus D. Feeding patterns in seagrass beds of three-spined stickleback Gasterosteus aculeatus juveniles at different growth stages. J Mar Biol Assoc United Kingdom. 2015; 1–9. doi:10.1017/S0025315415000569 |
| 46-66 | Main: Daphnia sp.  Simuliidae (mostly in June), Chironomidae (mostly in July),  Cladocera (mostly in May),  Complementary:  Copepoda, Ephemeroptera,  Lepidoptera, Heteroptera, Sporadically - fish eggs and fry. | May-August | Visual | Warta River,  Poland |  | freshwater | impounding river, submersed pond-weeds | Dukowska M, Grzybkowska M, Marszał L, Zięba G. The food preferences of three-spined stickleback, Gasterosteus aculeatus L., downstream from a dam reservoir. Oceanol Hydrobiol Stud. 2009;38: 39–50. doi:10.2478/v10009-009-0020-x |
| 29-76 | Cladocera  pelagic microcrustaceans, littoral cladocerns, amphipods, chironomids,  molluscs, ostracods, Trichoptera larvae | Autumn  Spring  Summer | Visual | Newfoundland lakes, Canada |  | freshwater | shallow oligotrophic lake | Campbell CE. Prey Selectivities of Threespine Sticklebacks (Gasterosteus-Aculeatus) and Phantom Midge Larvae (Chaoborus Spp) in Newfoundland Lakes. Freshw Biol. 1991;25: 155–167. doi:10.1111/j.1365-2427.1991.tb00481.x |
| 31 – 75 | Chironomids,  Copepods,  Cladocera,  Ostracods,  Rotifers,  Clams (Bivalvia),  Stickleback eggs | June-September | Visual | Karluk and Bare lake, Alaska |  | freshwater | lake | Greenbank J, Nelson P. Life history of the threespine stickleback Gasterosteus aculeatus Linnaeus in Karluk Lake and Bare Lake, Kodiak Island, Alaska. Fish Bull. 1959;59: 537–559 |
| 40 – 73  6 – 30  38 – 96 | Higher Crustacea,  Chironomids,  Copepoda,  Oligochaeta,  Cladocera,  Ostracoda  Cladocera,  Copepoda,  Chironomids  Ostracoda,  Rotifera,  Diatoms  Higher Crustacea,  Copepoda,  Diptera,  Sticklebacks eggs and larvae,  Annelida | monthly  August, June | Visual | Birket, UK  Easdale Quarry,  Argyll, UK |  | freshwater  brackish  3.6 psu | stream | Hynes H. The food of freshwater sticklebacks (Gasterosteus aculeatus and Pygosteuspungitius), with a review of methods used in studies of the food of fishes. J Anim Ecol. 1950;19: 36–58. doi:10.2307/1570 |
| 2 – 110 | Higher Crustacea,  Copepoda,  Cladocera,  Fish eggs and larvae,  Insects,  Ostracoda,  Polychaeta  Gastropoda |  | Visual | Denmark | Various Danish waters | brackish | *Zostera* regions | Blegvad, H. On the food of fish in the Danish waters within the Skaw. Rep. Danish Biol. 1917. Sta. 24: 19-72. |
| Adult  Juvenile | Copepoda,  Hemiptera,  Oligochaeta  Chironomidae,  Amphipoda,  Nematoda,  Fish eggs,  Bivalvia  Harpacticoida,  Calanoida  Cyclopoida,  Diatoms,  Rotifera,  Nematoda,  Oligochaeta,  Ostracoda,  Amphipoda | Spring  Summer  Autumn | Visual | St. Andrews,  New Brunswick,  Canada | 45°5′N  67°5′W | brackish  0 – 28 psu | tidal saltmarshes | Delbeek, J.C.; Williams DD. Food resource partitioning between sympatric populations of brackishwater sticklebacks. Journal of Animal Ecology. 1987. pp. 949–967. doi:10.2307/4959 |
| average length 12 ± 3 | Amphipods,  Zooplankton (copepods, ostracods) | Summer  July-August | Experiment | inner archipelago of the Askö area western Baltic Proper | 58°48’N, 17°40’ E | brackish  6.3 – 6.5 psu | sheltered bay, shallow (1.2 m deep) | Reiss K, Herriot MB, Eriksson BK. Multiple fish predators: Effects of identity, density, and nutrients on lower trophic levels. Mar Ecol Prog Ser. 2014;497: 1–12. doi:10.3354/meps10622 |
| Larvae  9.1±0.8 | Copepoda (Acartia spp., Eurytemora affinis)  Cladocera (Bosmina longispina)  Rotifera | July | Experiment | SW coast of Finland, the northern Baltic Sea |  | brackish  6 psu |  | Lehtiniemi M, Hakala T, Saesmaa S, Viitasalo M. Prey selection by the larvae of three species of littoral fishes on natural zooplankton assemblages. Aquat Ecol. 2007;41: 85–94. doi:10.1007/s10452-006-9042-6 |
