# Supplementary figures and images for: DNA metabarcoding reveals diverse diet of the three-spined stickleback in a coastal ecosystem
Source: PLoS One. 2017 Oct 23;12(10):e0186929. doi: 10.1371/journal.pone.0186929 (PMC5653352; doi:10.1371/journal.pone.0186929)

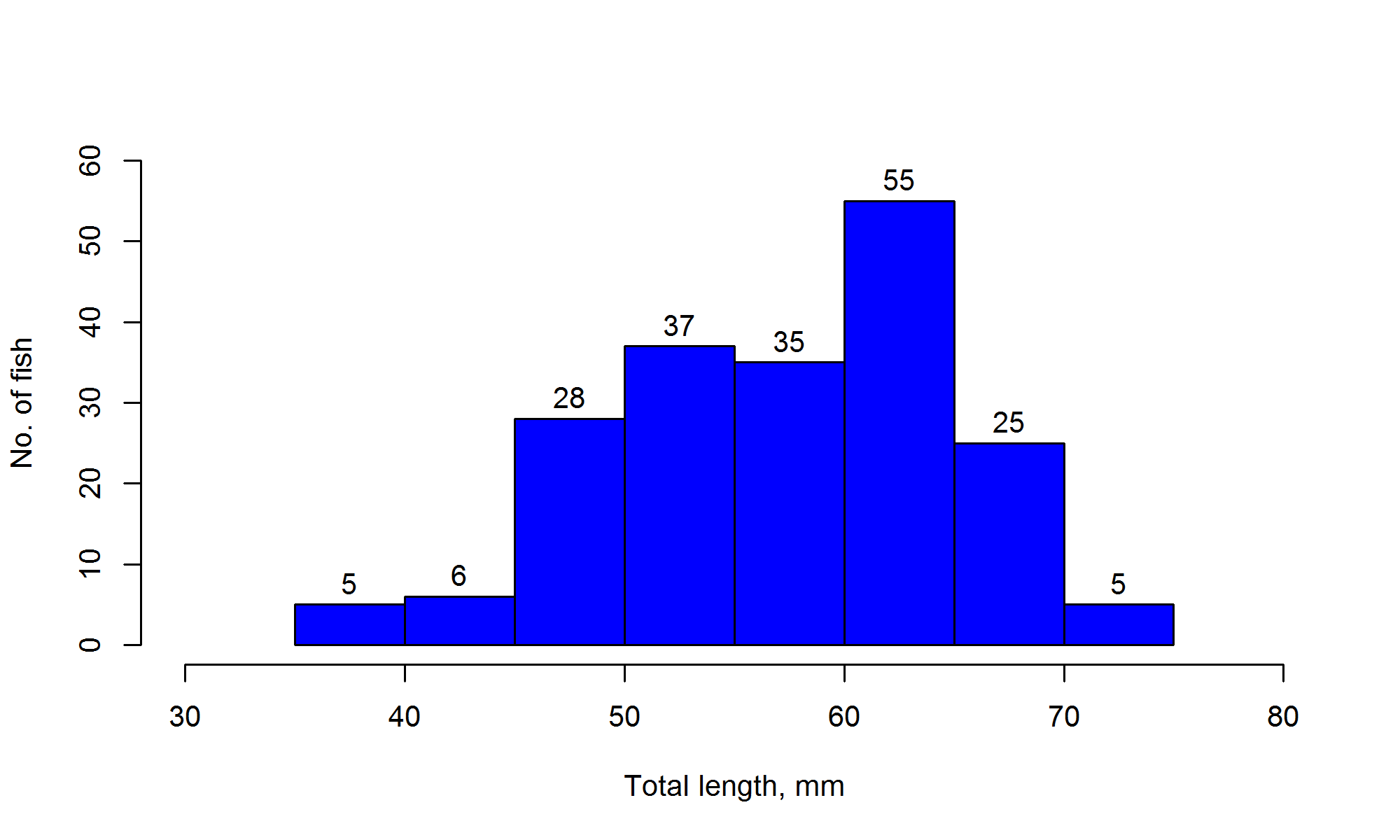

Supplement: S1 Fig — (TIF) [file pone.0186929.s004.tif]

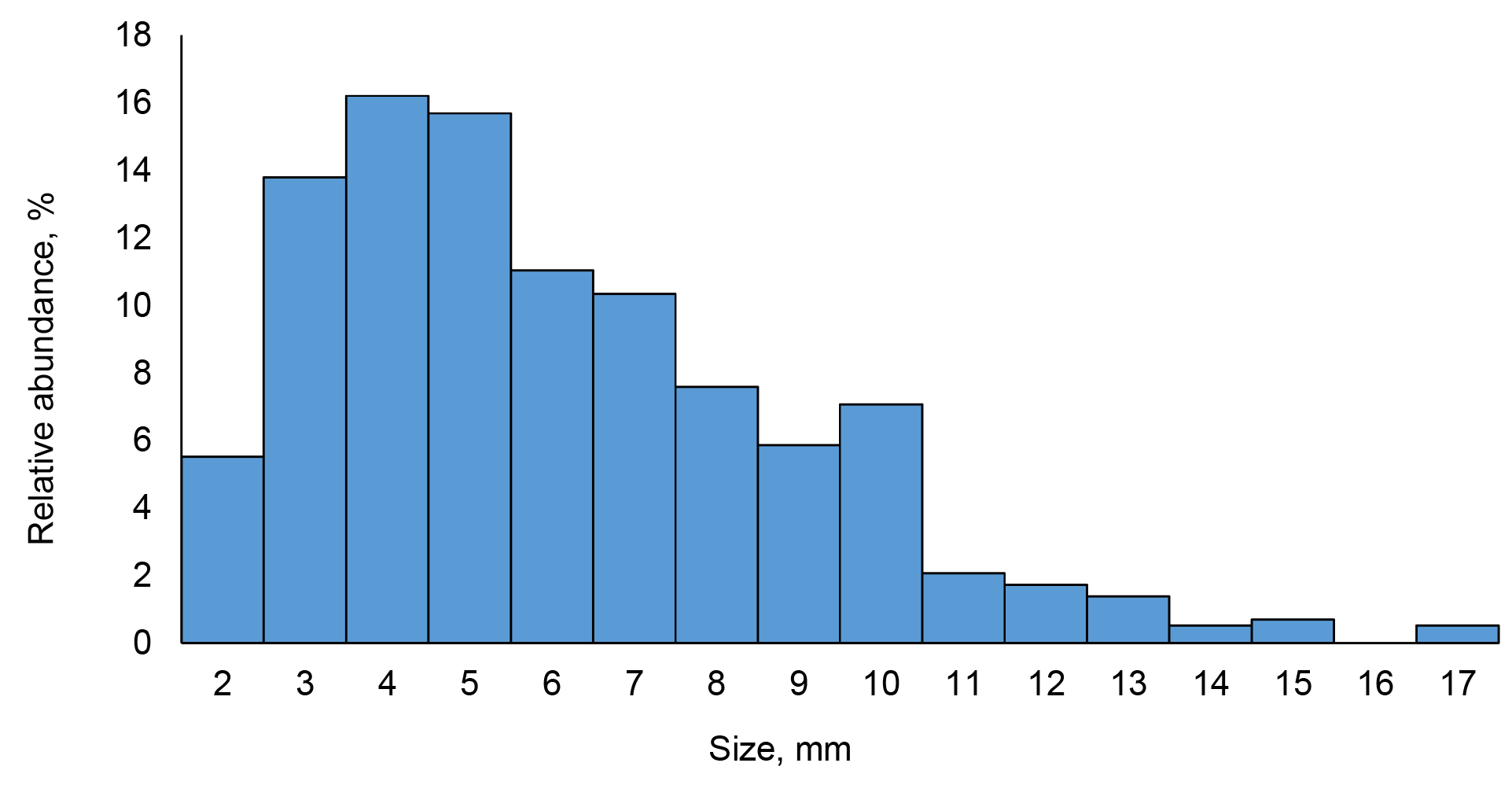

Supplement: S2 Fig — (TIF) [file pone.0186929.s005.tif]
