# Supplementary material for: DNA metabarcoding reveals diverse diet of the three-spined stickleback in a coastal ecosystem
Source: PLoS One. 2017 Oct 23;12(10):e0186929. doi: 10.1371/journal.pone.0186929 (PMC5653352; doi:10.1371/journal.pone.0186929)
Supplement: S1 Appendix — (DOCX) [file pone.0186929.s006.docx]

# Comparison of quantification from OTU reads and results of visual stomach content analysis

## Methods

After sequencing, we obtained an OTU table showing the number of reads per taxon found in the stomach of each fish. To estimate the relative abundance of a certain prey in the stomach, and to make data from different fish individuals comparable, numbers of reads were normalized to the total number of reads in each sample (individual), and proportions of different taxa in each stomach were estimated (hereafter termed as “%N_bar_”). Thus, proportions of prey in the stomachs are based on the number of OTU reads per taxon (%N_bar_) (e.g., [1]). Frequency of occurrence was also estimated - %Fbar, the percentage of stomachs in which a prey (OTU) was present.

## Results

As revealed by barcoding, Insecta (chironomids), Maxillipoda (harpacticoid copepods) and Branchiopoda (cladocerans) were the dominating food items, comprising 48%, 19% and 15% of all prey respectively (Fig A1). At the species level, the main prey were the chironomid *Tanytarsus usmaensis*, the harpacticoid *Tachidius discipes*, and the cladoceran *Pleopis polyphemoides* (Fig A2).

**Fig A1**. **Proportion of different classes in stomachs based on number of OTU reads (%N_bar_).** Only classes with >1% of OTU reads are shown.

**Fig A2. Main prey species (written) as indicated by relationship between relative abundance (%N_bar_, prey proportion in stomach based on number of OTU reads) and frequency of occurrence (%F_bar_).**

In general, the two methods used – barcoding and visual stomach content analysis - showed consistent patterns: at the population level, frequency of occurrence determined by visual analysis (%Fvis) correlated well with the proportions of prey in the stomachs based on the number of OTU reads (%Nbar; Fig A3), except for Bivalvia, which may have been underrepresented in the barcoding analysis.

**Fig A3. Diet of three-spined stickleback.** Relationship between the results of two methods used: proportions of prey in the stomachs based on the number of OTU reads (%N_bar_) and frequency of occurrence determined by visual analysis (%F_vis_).

## Discussion

Although read counts can be used as a semi-quantitative proxy for diet composition [1–4], several well-known issues still impede the use of DNA metabarcoding for quantification. Quantitative estimates of certain prey in the stomach may be influenced by prey size, level of digestion, DNA preservation, as well as experimentally introduced biases from DNA extraction, primer-template mismatches, PCR amplification bias, OTU clustering, reference library quality and taxonomic assignment process [5–12]. One way to reduce such biases is to introduce correction factors, by creating a library of mixed prey standards and then using them to correct counts from unknown composition [13]. This was not done in our study, but the application of alternative methods (visual analysis) enabled us, at least to some extent, to validate the DNA metabarcoding results (Fig A3).

## References

1. Soininen EM, Ravolainen VT, Bråthen KA, Yoccoz NG, Gielly L, Ims RA. Arctic Small Rodents Have Diverse Diets and Flexible Food Selection. PLoS One. 2013;8. doi:10.1371/journal.pone.0068128

2. Kowalczyk R, Taberlet P, Coissac E, Valentini A, Miquel C, Kamiński T, et al. Influence of management practices on large herbivore diet-Case of European bison in Białowieza Primeval Forest (Poland). For Ecol Manage. 2011;261: 821–828. doi:10.1016/j.foreco.2010.11.026

3. Deagle BE, Kirkwood R, Jarman SN. Analysis of Australian fur seal diet by pyrosequencing prey DNA in faeces. Mol Ecol. 2009;18: 2022–2038. doi:10.1111/j.1365-294X.2009.04158.x

4. Soininen EM, Valentini A, Coissac E, Miquel C, Gielly L, Brochmann C, et al. Analysing diet of small herbivores: the efficiency of DNA barcoding coupled with high-throughput pyrosequencing for deciphering the composition of complex plant mixtures. Front Zool. 2009;6: 16. doi:10.1186/1742-9994-6-16

5. Kress WJ, García-Robledo C, Uriarte M, Erickson DL. DNA barcodes for ecology, evolution, and conservation. Trends Ecol Evol. 2015;30: 25–35. doi:10.1016/j.tree.2014.10.008

6. Pompanon F, Deagle BE, Symondson WOC, Brown DS, Jarman SN, Taberlet P. Who is eating what: Diet assessment using next generation sequencing. Mol Ecol. 2012;21: 1931–1950. doi:10.1111/j.1365-294X.2011.05403.x

7. Troedsson C, Simonelli P, Nägele V, Nejstgaard JC, Frischer ME. Quantification of copepod gut content by differential length amplification quantitative PCR (dla-qPCR). Mar Biol. 2009;156: 253–259. doi:10.1007/s00227-008-1079-8

8. Deagle BE, Tollit DJ. Quantitative analysis of prey DNA in pinniped faeces: Potential to estimate diet composition? Conserv Genet. 2007;8: 743–747. doi:10.1007/s10592-006-9197-7

9. Kembel SW, Wu M, Eisen JA, Green JL. Incorporating 16S Gene Copy Number Information Improves Estimates of Microbial Diversity and Abundance. PLoS Comput Biol. 2012;8. doi:10.1371/journal.pcbi.1002743

10. Polz MF, Cavanaugh CM. Bias in template-to-product ratios in multitemplate PCR. Appl Environ Microbiol. 1998;64: 3724–3730.

11. Piñol J, Mir G, Gomez-Polo P, Agustí N. Universal and blocking primer mismatches limit the use of high-throughput DNA sequencing for the quantitative metabarcoding of arthropods. Mol Ecol Resour. 2015;15: 819–830. doi:10.1111/1755-0998.12355

12. Blanco-Bercial L, Cornils A, Copley N, Bucklin A. DNA Barcoding of marine copepods: assessment of analytical approaches to species identification. PLoS Curr. 2014; 1–40. doi:10.1371/currents.tol.cdf8b74881f87e3b01d56b43791626d2

13. Thomas AC, Deagle BE, Eveson JP, Harsch CH, Trites AW. Quantitative DNA metabarcoding: Improved estimates of species proportional biomass using correction factors derived from control material. Mol Ecol Resour. 2016;16: 714–726. doi:10.1111/1755-0998.12490
